# Role of Spinal Cord α_2_-Adrenoreceptors in Noradrenergic Inhibition of Nociceptive Transmission During Chemotherapy-Induced Peripheral Neuropathy

**DOI:** 10.3389/fnins.2019.01413

**Published:** 2020-01-15

**Authors:** José Tiago Costa-Pereira, Joana Ribeiro, Isabel Martins, Isaura Tavares

**Affiliations:** ^1^Unit of Experimental Biology, Department of Biomedicine, Faculty of Medicine, University of Porto, Porto, Portugal; ^2^Institute of Molecular and Cell Biology, University of Porto, Porto, Portugal; ^3^I3S-Institute for Investigation and Innovation in Health, University of Porto, Porto, Portugal

**Keywords:** descending pain modulation, antidepressants, paclitaxel, chemotherapy side-effects, cancer treatment, pain

## Abstract

Chemotherapy-induced peripheral neuropathy (CIPN) is a problem during cancer treatment and for cancer survivors but the central mechanisms underlying CIPN remain understudied. This study aims to determine if CIPN is associated with alterations of noradrenergic modulation of nociceptive transmission at the spinal cord. CIPN was induced in male Wistar rats by paclitaxel injections. One month after CIPN induction, the behavioral effects of the administration of reboxetine (noradrenaline reuptake inhibitor), clonidine (agonist of α_2_-adrenoreceptors; α_2__–_AR) and atipamezole (antagonist of α_2__–_AR) were evaluated using the von Frey and cold plate tests. Furthermore, we measured the expression of the noradrenaline biosynthetic enzyme dopamine-β-hydroxylase (DBH) and of α_2__–_AR in the spinal dorsal horn. Reboxetine and clonidine reversed the behavioral signs of CIPN whereas the opposite occurred with atipamezole. In the 3 pharmacological approaches, a higher effect was detected in mechanical allodynia, the pain modality which is under descending noradrenergic control. DBH expression was increased at the spinal dorsal horn of paclitaxel-injected animals. The enhanced noradrenergic inhibition during CIPN may represent an adaptation of the descending noradrenergic pain control system to the increased arrival of peripheral nociceptive input. A potentiation of the α_2__–_AR mediated antinociception at the spinal cord may represent a therapeutic opportunity to face CIPN.

## Introduction

Chemotherapy is the most common approach for cancer treatment but it frequently induces neuropathy. This chemotherapy-induced peripheral neuropathy (CIPN) is a clinical problem that may impose changes in cancer treatment and can persist after cessation of chemotherapy (Mantyh, 2006; Seretny et al., 2014). CIPN is characterized by multiple sensory features which include changes in pain responses, such as spontaneous pain, allodynia and hyperalgesia.

The neurotoxic mechanisms underlying CIPN depend on the cytostatic drug (Kerckhove et al., 2017). Paclitaxel, one of the most effective cytostatic drug, has been shown to cause CIPN in humans and in animal models (Polomano et al., 2001; Cavaletti and Marmiroli, 2010). Increases in nociceptive behavioral responses in paclitaxel-induced CIPN models were ascribed to peripheral fiber loss, mitochondrial swelling and vacuolization of peripheral axons and hyperexcitability of dorsal root ganglion neurons (Cliffer et al., 1998; Flatters and Bennett, 2006; Boehmerle et al., 2014; Yadav et al., 2015). The central changes during paclitaxel-induced CIPN remain understudied but since paclitaxel has a very low ability to cross the blood-brain barrier (Cavaletti and Marmiroli, 2010), it is likely that the central changes during paclitaxel-induced CIPN are caused by the neurotoxic effects triggered by the cytostatic at the periphery. At the spinal cord, paclitaxel-injected animals show increased spontaneous activity of wide-dynamic range neurons (Cata et al., 2006) and reduced local GABAergic tonic inhibition (Yadav et al., 2015). At the supraspinal level, the periaqueductal gray matter (PAG), a key area of the descending pain modulatory system, shows increases in spontaneous and evoked neuronal firing (Samineni et al., 2017). Furthermore, in a recent exploratory study using diffusion weighted magnetic resonance, alterations in the activity of the PAG were reported (Ferris et al., 2019). Using an animal model of paclitaxel-induced CIPN, we recently reported increases in the activity of serotoninergic neurons of the rostroventromedial medulla (RVM), an area that relays descending modulation from the PAG to the spinal cord (Costa-Pereira et al., 2019).

CIPN is empirically treated with antidepressant drugs which increase noradrenaline (NA) and serotonin (5-HT) levels (Sisignano et al., 2014), but the relative contribution of each neurochemical system is starting to be unraveled. Using the paclitaxel-induced CIPN model, we have recently described an involvement of 5-HT with an enhanced facilitatory effect of 5-HT3 receptors at the spinal cord (Costa-Pereira et al., 2019). Nothing is known about descending noradrenergic modulation in the paclitaxel-induced CIPN model. This is important since descending pain modulation has some distinct features between different preclinical models (Porreca et al., 2002; Ossipov et al., 2014). Furthermore descending modulatory systems are crucial to balance between inhibition (antinociception) and facilitation (pro-nociception) (Tracey and Mantyh, 2007; Heinricher et al., 2009). An imbalance of descending modulation toward facilitation was proposed to account for chronic pain installation. It is well established that NA release at the spinal dorsal horn is mainly originated from the pontine locus coeruleus (LC) (Pertovaara, 2006; Heinricher et al., 2009) and leads to analgesia by activating spinal α_2__*A*_-adrenoreceptors (α_2__*A*_-AR) which block the nociceptive transmission at the spinal dorsal horn, both pre and postsynaptically (Kawasaki et al., 2003; Pertovaara, 2013). Intrathecal administration of α_2_-AR agonists induces antinociception in humans and animal models whereas the opposite occurs with α_2_-AR antagonists (Eisenach et al., 1996; Budai et al., 1998). However, NA may trigger pain facilitation after brain release (Bie et al., 2003; Ortiz et al., 2008; Martins et al., 2015). Further adding complexity to the studies of noradrenergic pain modulation, an effect of the pain model was reported. In traumatic neuropathic pain models, noradrenergic upregulation occurs with increased spinal NA levels and enhanced potency of α_2_-AR (Ma and Eisenach, 2003; Bantel et al., 2005) but with the progression of traumatic neuropathy, a gradual loss of descending noradrenergic inhibition occurs (Hughes et al., 2013, 2015). In diabetic neuropathy the descending noradrenergic inhibition is impaired and NA exerts pain facilitation at the spinal level (Kinoshita et al., 2013). These results show that the specificities of each preclinical pain model should be considered in the studies of descending noradrenergic pain control.

To evaluate if descending noradrenergic modulation of spinal nociceptive transmission is altered during paclitaxel-induced CIPN, we used a validated model of paclitaxel-induced CIPN to study the noradrenergic modulation of nociceptive transmission at the spinal cord. We first evaluated the nociceptive behavioral effects of the administration of reboxetine (noradrenaline reuptake inhibitor), clonidine (agonist of α_2_-adrenoreceptors; α_2__–_AR) and atipamezole (antagonist of α_2__–_AR). Then we evaluated the immunohistochemical expression of the noradrenaline synthetizing enzyme, dopamine-β-hydroxylase (DBH), at the spinal dorsal horn. We analyzed the expression of α_2__–_AR at the spinal dorsal horn, using immunohistochemistry and western blot approaches.

## Materials and Methods

### Animals

Wistar male rats (weighing 175–190 g; Charles River, France) were housed in a 12/12 h light/dark environment at 22 ± 2°C and received food and water *ad libitum*. All behavioral experiments were conducted in the light phase. The animals were acclimated to the housing facility for a least 1 week before the onset of experiments. The animals were randomly housed in pairs and selected from the cage before each procedure.

The experiments were approved by the Animal Ethical Committee of the Faculty of Medicine of University of Porto and Directorate-General of Food and Veterinary Medicine–Portuguese National Authority for Animal Health (license 0421/000/000/2018) and performed in accordance with the European Community Council Directive (2010/63/EU) and the ethical guidelines of the International Association for the Study of Pain (IASP) in conscious animals (Zimmermann, 1983).

### Induction of the CIPN Model

The CIPN model was induced as described previously (Polomano et al., 2001). Briefly, paclitaxel (Taxol^®^) (2.0 mg Kg^–1^ – cumulative dose of 8.0 mg Kg^–1^) (Tocris, United Kingdom) was dissolved in a solution of 4% Dimethyl Sulfoxide (DMSO). Rats weighing 190-200 g received an intraperitoneal (i.p.) injection of the paclitaxel solution in 4 alternate days (day 1, 3, 5, and 7). The injections were performed between 9 a.m. and 11 a.m. Control animals were injected with 4% DMSO. The DMSO concentration was elected based on previous studies showing that it is the minimal concentration of DMSO required to resuspend paclitaxel and it does not induce any toxic effects (Worthley and Schott, 1969; Chen et al., 2011; Braz et al., 2015).

### Drug Delivery and Behavioral Evaluation

Three weeks after CIPN induction, all rats weighing 290–300 g underwent surgical implantation of sterile silicone catheter (0.31 × 0.64 × 0.17 mm; Freudenberg, Germany). Briefly, the animals were deeply anesthetized with medetomidine (0.25 mg Kg^–1^) and ketamine hydrochloride (60 mg Kg^–1^) and the catheter was inserted in the subarachnoid space in caudal direction until the tip reached the L4/L5 spinal cord segment. After surgery, the rats were single-housed to avoid interfering with cagemate’s catheter. The correct placement of the intrathecal catheter was confirmed after dissection. The few animals presenting severe signs of locomotor impairment after surgery were excluded from the study. The animals were then used in pharmacological experiments to test the behavioral nociceptive effects of reboxetine (NA reuptake inhibitor), atipamezole (α_2__*A*__–_AR blockade) and clonidine (α_2__*A*__–_AR agonist). All experiments were performed 30 days after the first paclitaxel injection.

#### Reboxetine Experiments

To study the effects of NA reuptake inhibition on nociceptive behaviors, we injected the selective reuptake inhibitor, reboxetine mesylate (10 mg Kg^–1^) (Tocris Bioscience, United Kingdom) (DMSO: *n* = 5; paclitaxel: *n* = 5) using the i.p. route. Reboxetine was dissolved in saline and the control groups were injected with saline (DMSO: *n* = 5; paclitaxel: *n* = 5). Previous study showed reboxetine did not affect motor performance (Lapmanee et al., 2013).

The effects of reboxetine on mechanical allodynia were evaluated before (T0) and at 30, 60, 120, and 240 min after injection. To evaluate mechanical nociceptive responses, we used the von Frey test as described previously in the CIPN model (Costa-Pereira et al., 2019). Briefly, the test was performed after 20 min acclimatization to equipment, according to the “up and down” method (Chaplan et al., 1994), which consists on the application of monofilaments between 0.4 and 26.0 g (Stoelting, United States) starting with the 2.0 g monofilament. Each animal was tested twice at an interval of 3–5 min, each value obtained was logarithmic transformed and averaged.

The effects of reboxetine on cold hyperalgesia were assessed at 30 min, the time that has been previously shown to be of maximum reboxetine (Hughes et al., 2015), which was further confirmed by the present results using the von Frey test. Cold responses were studied as described previously (Costa-Pereira et al., 2019) using the cold plate test. After a training period of 3 days for habituation purposes in the device, the animals were placed on the plate at 0°C and the withdrawal latency was recorded. The cut-off period of 60 s was applied to avoid any tissue damage.

#### Atipamezole Experiments

To evaluate the effects of the blockade of spinal α_2__*A*__–_AR on nociceptive behaviors, we administered the *α_2__*A*_*_–_*AR* antagonist atipamezole (Tocris Bioscience, United Kingdom) at 5 μg (DMSO: *n* = 5; paclitaxel: *n* = 4). Atipamezole was administered using the intrathecal route and was dissolved in 0.9% saline solution. Based on previous study (Dimitrov et al., 2013), we assessed the effects of atipamezole on mechanical and cold hypersensitivity 30 min after antagonist injection. Atipamezole did not induce any sedative effects (Pertovaara et al., 1994).

#### Clonidine Experiments

To assess the effects of the activation of spinal α_2__*A*__–_AR on nociceptive behaviors, we intrathecally administered the agonist clonidine (Sigma-Aldrich, United States) at 3 doses: 0.1 μg (DMSO: *n* = 6; paclitaxel *n* = 7), 1 μg (DMSO: *n* = 7; paclitaxel *n* = 7) or 10 μg (DMSO: *n* = 8; paclitaxel *n* = 6). Clonidine was dissolved in 0.9% saline solution and the respective control groups were injected with saline (DMSO: *n* = 7; paclitaxel *n* = 6). Mechanical and thermal hypersensitivity were evaluated before and 30 min after clonidine injection, which has previously been shown to be the time of the maximal drug effect (Yaksh et al., 1995). In order to evaluate possible sedative effects of the higher clonidine dose (10 μg), 2 additional animals were tested in the rotarod as described previously (Vanderah et al., 2001). Briefly, the test was performed using DMSO-injected animals after training once a day for three consecutive days. Training consisted on placing the rats on a rotating rod (Ugo Basile, Varese, Italy) with the rate of rotation set at 10 rpm, until they fell off or until reaching a cutoff time set at 180 s. The evaluated animals remained on the rod for 180 s which indicates that the animals did not have motor impairments after clonidine injection.

### Immunohistochemistry

Thirty days after the first paclitaxel injection, the rats were deeply anesthetized with an overdose of an i.p. injection of sodium pentobarbital (65 mg Kg^–1^) and perfused with 100 ml of calcium free Tyrode’s solution, followed by 750 ml of 4% paraformaldehyde in 0.1M phosphate buffer. The lumbar spinal cord segments were removed, immersed in a fixative for 4 h and cryopreserved in a 30% sucrose solution. The segments were then sliced at 30 μm in a freezing microtome and used for the DBH and α_2__*A*_-AR immunoreactions described below. The L4 and L5 sections were used for immunodetection of DBH (DMSO: *n* = 6; paclitaxel: *n* = 5) and α_2__*A*_- AR (DMSO: *n* = 5; paclitaxel: *n* = 5).

#### Dopamine-β-Hydroxylase (DBH) Immunoreaction

For the DBH-immunoreaction, one in every fourth spinal L4 and L5 sections were incubated with a monoclonal anti-DBH primary antibody (Millipore Catalogue No. MAB308) diluted at 1:5000, followed by a horse biotinylated anti-mouse secondary antibody (Dako, Denmark; 1:200). After several washes, the sections were incubated in PBS-T containing the avidin-biotin complex (1:200; ABC, Vector, United States). The bound peroxidase was revealed using 0.0125% 3,3′- diaminobenzidine tetrahydrochloride (DAB) (Sigma-Aldrich, United States). The immunodetection of DBH-immunoreactive fibers was assessed as described above.

The DBH labeled sections were observed using a light microscope (Axioskop 40 model, Zeiss^®^, Switzerland) coupled to a high-resolution digital camera (Leica EC3 model) and the LAS 4.6.0. software (Leica Microsystems^®^) and maintaining the same exposure and light settings. The quantification of DBH labeling of 3 randomly taken L4 and L5 spinal sections was performed on the ImageJ^®^ software (U. S. National Institutes of Health, United States) based on a method previously described (Hughes et al., 2013; Costa-Pereira et al., 2019). Briefly, the mean level of background was determined for each section and lamina using ROI analysis of small areas without DBH immunoreaction. The threshold level for DBH positive pixels was adjusted at a value of 5 standard deviations above the mean background level. The mean percentage of DBH positive pixels in laminae I-II, lamina III, IV, and V was then calculated.

The total DBH fibers length in each lamina of spinal dorsal horn were also calculated using a semi quantitative skeleton analysis adapted from Willing et al. (2017). Briefly, after an adjustment of the threshold level as abovementioned, the images were converted to binary images, skeletonized using the skeleton macro from ImageJ and evaluated in terms of number of pixels occupied by skeletons. The number of pixels were then converted to millimeter scale.

#### α_2__*A*_-AR Immunoreaction

For the α_2__*A*_-AR immunoreaction, one in every fourth spinal sections was incubated with a rabbit-raised anti-α_2__*A*_ primary antibody (Neuromics; Cat. No. RA14110), diluted at 1:500, followed by incubation for 1 h with a donkey anti-rabbit Alexa 488 (Molecular Probes^®^; 1:1000). Photomicrographs were taken under the same time exposure, capture parameters and laser light wavelength (488 nm) on an ApoTome Slider (Zeiss^®^) fluorescence microscope coupled to the AxioVision Rel. 4.8. software (Zeiss^®^). The images were analyzed in order to calculate the percentage of pixels occupied by α_2__*A*_-AR immunoreactivity, size and number of α_2__*A*_-AR positive neurons in the spinal dorsal horn of 5 randomly taken sections using the ROI manager. The mean percentage of α_2__*A*_-AR positive pixels in laminae I-II was automatically calculated by the ImageJ software. The size and number of α_2__*A*_-AR positive neurons were also automatically quantified using the “Analyze Particles” function of ImageJ software.

### Western- Blot Analysis of α_2__*A*_ AR

The dorsal portion of the L4 and L5 segments from DMSO (*n* = 5) and paclitaxel-injected animals (*n* = 5) were homogenized with lysis buffer (TBS-T: 20 mM Tris HCl pH 7.4; 150 mM NaCl; 0.1% Triton X-100) containing phosphatase inhibitors and protease inhibitor. A total of 20 μg of protein was loaded and electrophoresed on 12% SDS-PAGE. The proteins were then electroblotted onto nitrocellulose membranes. After incubation with 5% of Blotting-Grade Blocker (Bio-Rad, United States), the membrane was incubated with rabbit anti-α*_2__*A*_*-ARs (Neuromics; Cat. No. RA14110) diluted at 1:1000, followed by an anti-rabbit secondary antibody conjugated to horseradish peroxidase (HRP) (1:10000; Jackson Immunoresearch Europe, United Kingdom). The immunoreactive bands were detected by Chemidoc system (Bio-Rad, United States). Glyceraldehyde 3-phosphate dehydrogenase (GAPDH) was used as loading protein internal control, with the membranes being incubated with mouse anti-GAPDH (1:10000; Abcam, United Kingdom) followed by incubation in anti-mouse secondary antibody conjugated to HRP (1:10000; Jackson Immunoresearch Europe, United Kingdom). Semi-quantification of bands was performed using Image Lab software (Bio-Rad, United States) and expressed in arbitrary units. The results of the quantification of α_2__*A*_-AR expression were presented as normalized for GAPDH.

### Specificity of Primary Antibodies

The specificity of the primary anti-DBH antibody was previously demonstrated (Howorth et al., 2009). We tested the specificity of alpha2AR using Western blot of liver samples since the liver tissue does not express mRNA for the receptor (Handy et al., 1993). No band was detected in the blot.

### Statistical Analysis

The behavioral results obtained in the Von Frey and cold plate tests were analyzed by two-way repeated measures of ANOVA followed by Tukey’s *post hoc* for multiple comparisons. The analysis of the percentage of change obtained in the von Frey and cold plate tests were conducted by ordinary two-way ANOVA followed by Tukey’s *post hoc* for multiple comparisons. DBH and α_2__*A*_-ARs expression in DMSO- and paclitaxel-injected animals were compared by unpaired *t*-test. Statistical analysis was performed by GraphPad Prism (GraphPad Software, United States). Data are presented as mean ± SD. *P* < 0.05 was considered statistically significant values.

## Results

In order to evaluate if increasing NA levels or interfering with the function of α_2__–_AR differentially affect nociceptive behavioral responses of control and paclitaxel- injected animals, we administered reboxetine, atipamezole or clonidine.

### Antinociceptive Effects of Reboxetine

**FIGURE 1:**
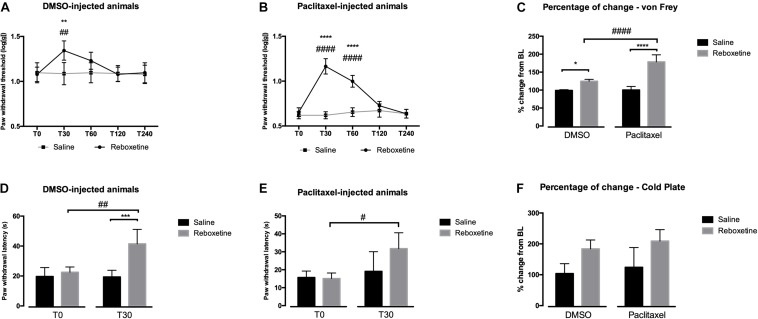
Nociceptive behavioral effects of the administration of reboxetine (30 days after the first paclitaxel injection). **(A,B)** Show a time-course analysis of paw withdrawal thresholds in the von Frey test of DMSO- and paclitaxel-injected rats, respectively, after saline or reboxetine administration. **(D,E)** Show the paw withdrawal latency in the cold plate test at baseline (T0) and 30 min (T30) after saline or reboxetine administration. Data are presented as mean ± SD. ^∗^*p* < 0.05; ^∗∗^*p* < 0.01; ^∗∗∗^*p* < 0.001; ^****^p < 0.0001 vs. saline; ^#^*p* < 0.05; ^##^*p* < 0.01; ^####^*p* < 0.001 vs. baseline (T0). Graphs **(C,F)** show the percentage of change from baseline of paw withdrawal thresholds and latencies after saline or reboxetine injection in the von Frey **(C)** and cold plate **(F)** tests. Data are presented as mean ± SD (saline: DMSO and paclitaxel *n* = 5; reboxetine at 10 mg/kg: DMSO and paclitaxel *n* = 5). ^∗^*p* < 0.05; ^****^p < 0.0001 vs. saline; ^####^*p* < 0.001 vs. DMSO.

In order to evaluate the behavioral nociceptive effects of an increase of NA levels, we injected the selective NA reuptake inhibitor reboxetine 30 days after the first paclitaxel administration. The results of paclitaxel administration in mechanical and cold sensitivities are shown in Figure 1. The analysis of the effects of reboxetine in the von Frey test (Figures 1A–C), showed that in DMSO-injected animals (Figure 1A) there was a significant interaction between treatments (saline vs. reboxetine) and time [*F*(4,32) = 17.60, *p* < 0.0001]. Reboxetine significantly increased paw withdrawal thresholds at 30 min after injection compared to saline (*p* = 0.0091) and before the injection (T0; *p* = 0.0064). The paw withdrawal thresholds returned to the baseline values 60 min after injection of reboxetine. The analysis of the effects of reboxetine in paclitaxel-injected animals (Figure 1B) revealed a significant interaction between treatment and time [*F*(4,32) = 62.73, *p* < 0.0001]. Reboxetine significantly increased paw withdrawal thresholds at 30 and 60 min after injection compared with saline and with T0 (*p* < 0.001). Paw withdrawal thresholds returned to the baseline values 120 min after injection of reboxetine. The injection of saline did not affect the behavioral responses of DMSO- and paclitaxel-injected animals (Figures 1A,B). Overall, the analysis of the variation from baseline, 30 min after injection of reboxetine or saline, revealed a significant interaction between treatments and experimental groups (DMSO *vs.* paclitaxel) [*F*(1,16) = 27.53; *p* < 0.0001; Figure 1C]. The increase of withdrawal thresholds was higher after reboxetine than after saline injection both in the DMSO- (*p* = 0.0116) and paclitaxel-group (*p* < 0.0001). The injection of reboxetine induced significantly higher percentages of change in the paclitaxel-group (178.75 ± 19.50%) than in DMSO-group (124.62 ± 5.23%; *p* < 0.0001).

The analysis of the effects of reboxetine in the cold plate test is shown in Figures 1D–F. In DMSO-injected animals (Figure 1D), reboxetine induced a significant interaction between treatments and time [*F*(1,8) = 23.12, *p* = 0.0013]. Reboxetine significantly increased paw withdrawal latency compared to saline injection (*p* = 0.0002) and T0 (*p* = 0.0012). The analysis of the effects of reboxetine in paclitaxel-injected animals (Figure 1E) revealed a significant interaction between treatments and time [*F*(1,8) = 5.466, *p* = 0.0476]. Reboxetine significantly increased the paw withdrawal latency compared to T0 (*p* = 0.0127). No statistically significant alterations in paw withdraw latency were induced after saline injection in DMSO- and paclitaxel-injected animals (Figures 1D,E). The overall analysis of the variation (Figure 1F) only revealed an effect of treatment [*F*(1,16) = 18.60, *p* = 0.0005] but no effects of the group [*F*(1,16) = 1.404, *p* = 0.2533] or interaction [*F*(1,16) = 0.017, *p* = 0.8969] which indicates that reboxetine increased significantly withdrawal latencies in both experimental groups compared with saline but without significant differences between the group (DMSO: 183.92 ± 28.77%; paclitaxel: 209.09 ± 37.09%).

### Pronociceptive Effects of Atipamezole

**FIGURE 2:**
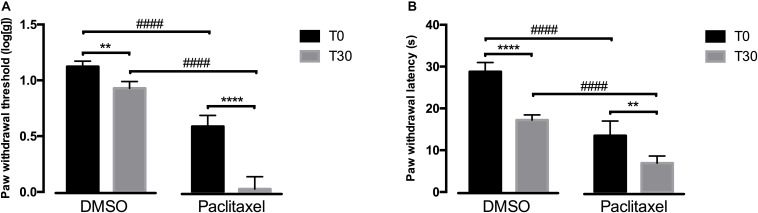
Nociceptive behavioral effects after the administration of atipamezole (30 days after the first paclitaxel injection). **(A)** Shows paw withdrawal threshold in the von Frey test at baseline (T0) and 30 min (T30) after atipamezole administration. **(B)** Shows paw withdrawal latency in the cold plate test at T0 and T30 after atipamezole administration. Data are presented as mean ± SD (DMSO *n* = 5; paclitaxel *n* = 4). ^∗∗^*p* < 0.01; ^****^p < 0.0001 vs. baseline (T0); ^####^*p* < 0.0001 vs. DMSO.

Since reboxetine administration had an effect at behavioral responses, we then evaluated if blocking α_2__*A*_-AR at the spinal cord by intrathecal administration of atipamezole, an α_2__*A*_-AR antagonist, also affected nociceptive behavioral responses. The behavioral effects of intrathecal are shown in Figure 2. Overall, the analysis of the effects of atipamezole in the von Frey test (Figure 2A) showed a significant interaction between groups (DMSO *vs.* paclitaxel) and time [*F*(1,7) = 57.37, *p* = 0.0001]. In comparison with the respective values at T0, the administration of atipamezole decreased paw withdrawal thresholds in the DMSO (*p* = 0.0087) and paclitaxel group (*p* < 0.0001). After atipamezole injection, the paw withdrawal thresholds of paclitaxel-injected animals were significantly lower than DMSO-treated animals (*p* < 0.0001). At baseline, the experimental groups were statistically different (*p* < 0.0001).

The analysis of the effects of atipamezole in the cold plate test (Figure 2B) showed a significant interaction between groups and time [*F*(1,7) = 8.048, *p* = 0.0252]. Compared to baseline, atipamezole significantly decreased paw withdrawal latencies in the DMSO (*p* < 0.0001) and paclitaxel groups (*p* = 0.0051). In the DMSO group, atipamezole decreased withdrawal latencies to values similar to those of the paclitaxel group at T0 (*p* = 0.1105; Figure 2B). Paw withdrawal latencies in the paclitaxel group were significantly lower than in the DMSO group after atipamezole injection (*p* < 0.0001). At T0, the DMSO- and paclitaxel groups were statistically different (*p* < 0.0001).

### Antinociceptive Effects of Clonidine

**FIGURE 3:**
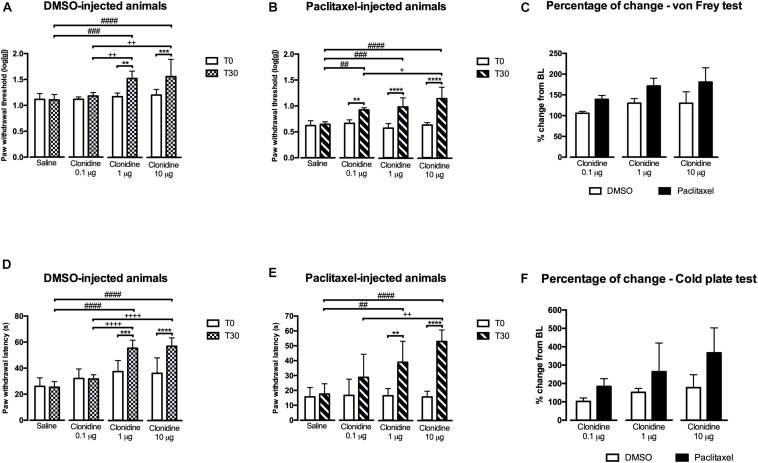
Nociceptive behavioral effects of the administration of clonidine (30 days after the first paclitaxel injection). **(A,B)** Show paw withdrawal thresholds in the von Frey test at baseline (T0) and 30 min (T30) after saline or clonidine administration. **(D,E)** Show paw withdrawal latencies in the cold plate test at T0 and T30 after saline or clonidine administration. **(C,F)** Show the percentage of change from baseline of paw withdrawal thresholds and latencies, in the von Frey **(C)** and cold plate **(D)** tests. Data are presented as mean ± SD (saline: DMSO *n* = 7; paclitaxel *n* = 6; clonidine at 0.1 μg: DMSO *n* = 6; paclitaxel *n* = 7; clonidine at 1 μg: DMSO *n* = 7; paclitaxel *n* = 7; clonidine at 10 μg: DMSO *n* = 8; paclitaxel *n* = 6). ^∗∗^*p* < 0.01; ^∗∗∗^*p* < 0.001; ^****^p < 0.0001 vs. baseline (T0); ^##^*p* < 0.01; ^###^*p* < 0.001; ^####^*p* < 0.0001 vs. saline. ^+^*p* < 0.05; ^++^*p* < 0.01; ^++++^*p* < 0.0001 vs. clonidine 0.1 μg.

Based on the results obtained with reboxetine and atipamezole, we then evaluated if activating α_2__*A*_-AR at the spinal cord by intrathecal administration of 3 doses of clonidine, an α_2__*A*_-AR agonist, also affected nociceptive behavior. The results of the study of the effects of 3 clonidine doses (0.1, 1, and 10 μg) are shown in Figure 3. In the von Frey test, the analysis of the effects of clonidine in DMSO-injected animals (Figure 3A) revealed a significant interaction between treatments and time [*F*(3,24) = 7.589, *p* = 0.0010]. The lower dose of clonidine (0.1 μg) produced no effects compared to T0 and saline. The higher doses of clonidine significantly increased paw withdrawal thresholds compared to T0 (1 μg: *p* = 0.003; 10 μg: *p* = 0.001), saline (1 μg: *p* = 0.0003; 10 μg: *p* < 0.0001), and clonidine at 0.1 μg (1 μg: *p* = 0.0077; 10 μg: *p* = 0.0015). No differences were detected between clonidine at 1 and 10 μg. Saline injection produced no significant effects and no differences were detected between the animals at baseline. The effects of clonidine in paclitaxel-injected animals in the von Frey test (Figure 3B) revealed a significant interaction between treatment and time [*F*(3,22) = 16.63, *p* < 0.0001]. The 3 doses of clonidine significantly increased paw withdrawal thresholds compared to baseline (0.1 μg: *p* = 0.0030; 1 μg: *p* < 0.0001; 10 μg: *p* < 0.0001) and saline (0.1 μg: *p* = 0.0020; 1 μg: *p* = 0.0001; 10 μg: *p* < 0.0001). Clonidine at 10 μg, showed higher withdrawal thresholds compared to clonidine at 0.1 μg (*p* = 0.0257; Figure 3B). No differences were detected between clonidine 0.1 and 1 μg neither between clonidine 1 and 10 μg. At T0, the paw withdrawal thresholds were not significantly different between the different conditions (saline and 3 doses of clonidine). The analysis of the variation from baseline (Figure 3C) showed an effect of experimental group [*F*(1,35) = 43.29; *p* < 0.0001] and treatment [*F*(2,35) = 10.13; *p* = 0.0003] but no interaction [*F*(2,35) = 0.613; *p* = 0.5473]. The 3 doses of clonidine significantly increased withdrawal thresholds in the paclitaxel group compared with DMSO group. Overall, the effects of the lower dose (0.1 μg) were significantly lower than the intermediate (1 μg; *p* = 0.0042) and the higher dose (10 μg; *p* = 0.0030). No statistically significant differences were detected between the doses of 1 and 10 μg.

In the cold plate test, the effects of clonidine in DMSO-injected animals (Figure 3D) showed a significant interaction between treatment and time [*F*(3,24) = 24.19, *p* < 0.0001]. The lower dose of clonidine (0.1 μg) produced no effects compared to T0 and saline. The higher doses of clonidine significantly increased paw withdrawal latency compared to T0 (1 μg: *p* = 0.0009; 10 μg: *p* < 0.0001), to saline (1 μg: *p* < 0.0001; 10 μg: *p* < 0.0001) and to clonidine at 0.1 μg (1 μg: *p* < 0.0001; 10 μg: *p* < 0.0001). No differences were detected between clonidine at 1 and 10 μg. Saline injection produced no significant effects and no differences were detected between the animals at baseline. The analysis of the effects of clonidine in paclitaxel-injected animals in the cold test (Figure 3E) revealed a significant interaction between treatments and time [*F*(3,44) = 7.087, *p* = 0.0005]. The lower dose of clonidine (0.1 μg) produced no effects compared to T0 and saline. The higher doses of clonidine significantly increased paw withdrawal latency compared to baseline (1 μg: *p* = 0.0025; 10 μg: *p* < 0.0001) and saline (1 μg: *p* = 0.0076; 10 μg: *p* < 0.0001). Clonidine at 10 μg showed higher withdrawal latencies compared to clonidine at 0.1 μg (*p* = 0.001; Figure 3E). No differences were detected between clonidine 1 and 10 μg (Figure 3E). Overall, the analysis of the variation from baseline (Figure 3F) revealed an effect of the experimental group [*F*(1,35) = 20.07; *p* < 0.0001] and treatment [*F*(2,35) = 6.689; *p* = 0.0035] but no interaction [*F*(2,35) = 1.257; *p* = 0.2971]. Therefore, the variation is higher in paclitaxel- than DMSO-injected animals. Overall the effects of the lower dose (0.1 μg) are significantly lower than the higher dose (10 μg; *p* = 0.0079).

### DBH-Immunoreaction at the Spinal Cord

**FIGURE 4:**
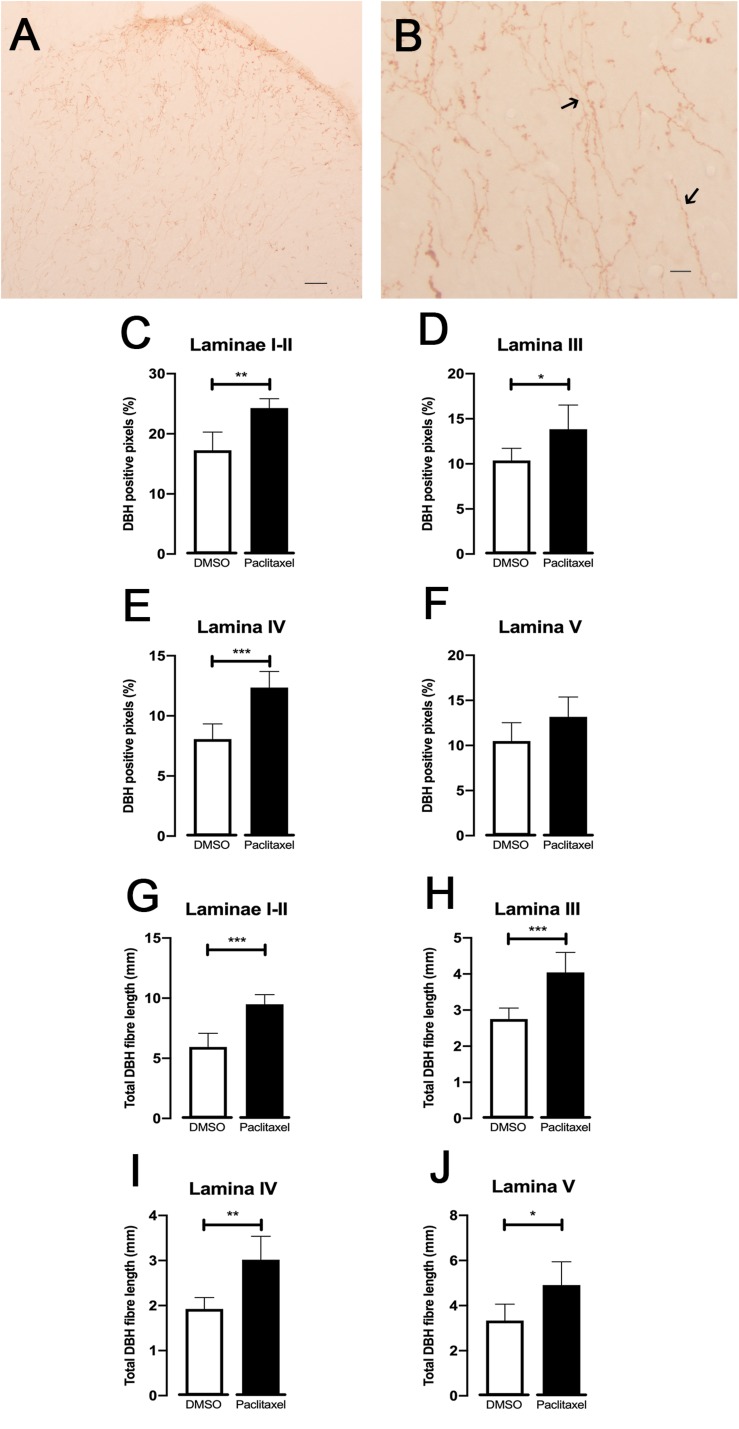
Spinal noradrenergic system evaluated by DBH immunoreaction (30 days after the first paclitaxel injection). Representative photomicrographs of DBH expression at the spinal dorsal horn **(A,B).** The details of fibers namely varicosities are better depicted in **(B)** (arrows). Scale bar in **(A,B)** 200 and 25 μm, respectively. The mean percentages of DBH positive pixels evaluated using the ROI manager are shown in laminae I-II **(C)**, III **(D)**, IV **(E),** and V **(F).** The total length of DBH-immunoreactive fibers quantified using skeleton macro is shown in laminae I-II **(G)**, III **(H)**, IV **(I),** and V **(J).** Data are presented as mean ± SD (DMSO *n* = 6; paclitaxel *n* = 6). ^∗^*p* < 0.05; ^∗∗^*p* < 0.01; ^∗∗∗^*p* < 0.001 paclitaxel vs. DMSO.

To evaluate if the noradrenergic innervation of the spinal dorsal horn is affected during CIPN, the DBH expression was analyzed (Figure 4). DBH-immunoreactive fibers were clearly recognized by the brown axons and varicosities scattered throughout the spinal dorsal horn (Figures 4A,B). The sections from paclitaxel-injected animals presented significantly higher percentages of DBH-positive pixels compared to the DMSO-injected animals in laminae I–II (*p* = 0.0011; Figure 4C), lamina III (*p* = 0.0216; Figure 4D), and IV (*p* = 0.0004; Figure 4E). No statistically significantly differences were detected in lamina V (*p* = 0.0652; Figure 4F). The total length of DBH-positive fibers was also analyzed (Figures 4G–J). Paclitaxel-injected animals showed longer DBH-positive fibers compared to DMSO-injected animals in laminae I–II (*p* = 0.0002; Figure 4G), lamina III (*p* = 0.0008; Figure 4H), IV (*p* = 0.0013; Figure 4I), and V (*p* = 0.0158; Figure 4J).

### Spinal Expression of α_2__*A*_-AR

**FIGURE 5:**
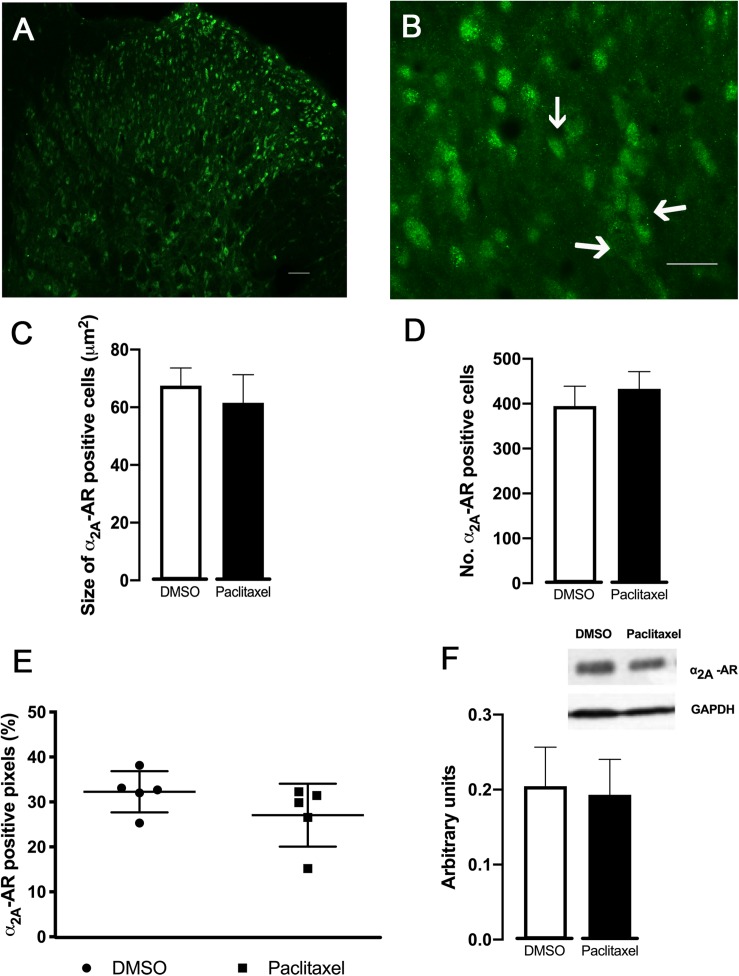
Expression of α_2__*A*_-AR in the spinal dorsal horn (30 days after the first paclitaxel injection). Representative photomicrographs of a α_2__*A*_-AR positive pixels at laminae I–II are shown in **(A,B)**. The immunolabelling occurs in structures with neuronal morphology, which are better represented by arrows in **(B)**. Scale bar in **(A,B)**: 100 and 50 μm, respectively. **(C,D)** Show the size and the number of α_2__*A*_-AR positive neurons, respectively, determined in laminae I-II in DMSO- and paclitaxel-injected animals. **(E)** Represents the percentage of α_2__*A*_-AR positive pixels in laminae I-II of DMSO- and paclitaxel-injected animals. Data are presented as mean ± SD (DMSO *n* = 5; paclitaxel *n* = 5). **(F)** Depicts the quantification of α_2__*A*_–AR assessed by western-blotting from DMSO- and paclitaxel-injected animals. GAPDH was used as a control of the western-blotting procedure. Data are presented as mean ± SD (DMSO *n* = 5; paclitaxel *n* = 5).

In order to evaluate if the higher effects of α_2__*A*_-AR ligands observed in paclitaxel-injected animals were related to changes in receptor expression, the analysis of the α_2__*A*_-AR expression at the spinal dorsal horn was performed (Figure 5). The immunofluorescence quantification of the superficial dorsal horn (laminae I-II) (Figure 5E) revealed that the percentage of α_2__*A*_-AR positive pixels were not statistically significant different between DMSO- and paclitaxel-injected animals (*p* = 0.2017). Likewise, the analysis of size and number of α_2__*A*_-AR positive objects did not show significantly differences between experimental groups (*p* = 0.2804 and *p* = 0.1820, respectively; Figures 5C,D).

The western-blotting analysis (Figure 5F) did not show statistically significant differences between DMSO- and paclitaxel-injected animals (*p* = 0.7174).

## Discussion

The present study shows, for the first time, that the noradrenergic modulation of spinal nociceptive transmission is altered in an animal model of paclitaxel-induced CIPN. This is relevant since the study of descending pain modulation should take into account the specific features of the preclinical model (Porreca et al., 2002; Ossipov et al., 2014) and descending noradrenergic modulation was never studied in the paclitaxel-induced CIPN model. To study noradrenergic modulation of nociceptive transmission at the spinal cord in paclitaxel-induced CIPN, we performed behavioral and structural studies. Regarding the behavioral studies, paclitaxel-injected animals treated with the selective NA reuptake inhibitor reboxetine showed a decrease of nociceptive behaviors. The antinociceptive effects of reboxetine were much more robust in paclitaxel-injected animals than in controls, both in magnitude and duration. Considering the increased NA levels in the spinal cord after reboxetine injection (Nakajima et al., 2012), the present findings showing antinociceptive effects after reboxetine administration indicate an increased recruitment of descending noradrenergic pain inhibition during paclitaxel-induced CIPN. The reboxetine data are further supported by the results of the intrathecal administration of the α_2__*A*_-AR antagonist atipamezole and of the α_2__*A*_-AR agonist clonidine since both drugs induced higher behavioral effects in paclitaxel-injected animals. The results suggest a relation between the dose and the magnitude of behavioral antinociceptive responses. Furthermore, the lowest clonidine dose (0.1 μg) failed to show an effect in DMSO-injected animals whereas in paclitaxel-injected animals it induced an antinociceptive effect. The clonidine data reinforce the hypothesis that in paclitaxel-induced CIPN there is an increased recruitment of noradrenergic modulation at the spinal cord. Incidentally, it should be noted that the current pharmacological studies support the existence of tonic noradrenergic inhibition since the intrathecal administration of atipamezole and clonidine to DMSO-injected animals altered their behavioral nociceptive responses. The existence of a tonic noradrenergic inhibition is a question under dispute since some studies show that atipamezole induces mechanical and cold allodynia in control animals (Xu et al., 1999), whereas others failed to show effects (Wei and Pertovaara, 2006; Patel et al., 2018). The role of the descending noradrenergic system during chronic pain installation is interesting. In traumatic pain models, some studies reported plastic changes and functional upregulation of spinal α_2_-AR (Stone et al., 1999; Ma and Eisenach, 2003; Bantel et al., 2005; Hayashida et al., 2008). This was proposed to compensate the enhanced nociceptive peripheral input, through increased noradrenaline levels and enhancement of the potency of α_2_-AR at the spinal cord. With the progression of traumatic neuropathy, a gradual loss of descending noradrenergic inhibition occurs along with an increase of descending facilitation (Viisanen and Pertovaara, 2007; Rahman et al., 2008; Hughes et al., 2013, 2015; Patel et al., 2018). By studying paclitaxel-induced CIPN using a short duration, the present result indicates that CIPN is similar to short-term models of traumatic neuropathy. Since the duration of neuropathy also affects the features of descending modulation, we will increase the duration of our studies in the paclitaxel-induced CIPN model to evaluate if a switch of descending noradrenergic modulation from inhibitory to facilitatory could account for the intensification of CIPN after long term chemotherapy treatments, a current problem for cancer survivors (Mantyh, 2006; Seretny et al., 2014).

As to the structural data, the levels of DBH in the spinal dorsal horn cord were higher in paclitaxel-injected animals. The analysis of the laminar distribution in what concerns the length of fibers and positive-pixels showed that the increase was due to contribution of all the spinal laminae. The increase in the DBH levels at the spinal cord matches the results obtained in traumatic neuropathic pain models (Ma and Eisenach, 2003) and indicates increased noradrenergic innervation of the spinal dorsal horn in chronic pain situations. The higher potency of atipamezole and clonidine in paclitaxel-injected animals cannot be explained by an increase in the expression of α_2__*A*_-AR at the superficial dorsal horn (laminae I–II), as no differences were found in the expression of α_2__*A*_-AR between paclitaxel- and DMSO-injected animals. An increase of potency of α_2_-AR without changes in receptor expression may be due to increased efficiency of G-protein coupled α_2_-AR (Bantel et al., 2005; Chen et al., 2007). The antinociceptive effects of clonidine are well reported in clinical studies (Rauck et al., 2015) and in models of traumatic peripheral nerve injury (Hayashida et al., 2008). Clonidine acts at presynaptic α_2_-AR located on the central terminals of primary afferents and on postsynaptic dorsal horn α_2_-AR (Pan et al., 2002; Mitrovic et al., 2003). In the present study, we cannot discriminate between the pre- and postsynaptic components of α_2_-AR. Paclitaxel induces abnormal outgrowth of primary afferent sensory neurons (Letourneau and Ressler, 1984; Cliffer et al., 1998) but since no changes in α_2_-AR expression were detected in the present study, it is likely that the primary afferents which express α_2_-AR are not structurally affected during CINP. The neurochemical nature of primary affect fibers damaged by paclitaxel is starting to be studied with indications that NMDA receptors are affected (Xie et al., 2016; Chen et al., 2019). Another emergent issue is the cell type which expresses α_2_-AR since at the spinal cord the receptors are present in neurons and glial cells (Xu et al., 2010). No differences were detected in the number and size of cell profiles immunostained for α_2_-AR between the experimental groups and most of the immunostained profiles were large and similar to neurons. Detailed comparisons of the expression of α_2_-AR in neurons and glial cells (astrocytes and microglia) between control and paclitaxel-injected animals can provide evidence about a possible role of spinal glia during CIPN. The behavioral studies with reboxetine, atipamezole and clonidine clearly showed more pronounced drug effects in mechanical allodynia than in cold hyperalgesia. This is an interesting finding since mechanical allodynia is a sensory modality predominantly modulated supraspinally, namely by brainstem centers engaged in noradrenergic inhibitory control (Xu et al., 1999; Saade et al., 2006; Hughes et al., 2015). The LC is likely to be the main source of the increased descending input since it is, by large, the main source of noradrenergic fibers at the spinal cord in the rat strain used in the present study (Tavares et al., 1997). Noradrenergic modulation from the LC is an important coordinator of the balance between inhibition and facilitation of descending pain control (Rahman et al., 2008; Martins et al., 2015). We showed antinociceptive effects of NA in paclitaxel-injected animals which indicates that during CIPN noradrenergic modulation from the LC preserves its inhibitory tone. We have recently shown that in paclitaxel-induced CIPN there is an increased activation of serotoninergic RVM neurons (Costa-Pereira et al., 2019). Since the RVM targets the LC and this connection is relevant for descending noradrenergic pain modulation (Sim and Joseph, 1992; Bahari and Meftahi, 2019) it is possible that the activation of RVM neurons recently reported in the paclitaxel-induced CIPN model (Costa-Pereira et al., 2019) is a trigger of increased recruitment of noradrenergic descending modulation during CIPN.

Patients affected by CIPN are empirically treated with antidepressant drugs which potentiate the effects of NA and serotonin but the relative contribution of each neurochemical system is unknown (Sisignano et al., 2014). By showing that in the paclitaxel CIPN model, the inhibitory function of the noradrenergic system is potentiated and based on our recent demonstration that the serotoninergic system exerts pronociceptive effects mediated by spinal 5HT3 receptors (Costa-Pereira et al., 2019), we propose that treating CIPN with drugs that mainly target the noradrenergic system may be a valuable approach in the future of cancer treatment. However, further preclinical studies are necessary namely in what concerns the evaluation of the mechanisms of noradrenergic modulation of nociceptive transmission during CIPN in female animals. Although no differences in the sex differences in mechanical allodynia were detected between male and female rodents (Hwang et al., 2012; Naji-Esfahani et al., 2016), one study showed sex differences in cold allodynia (Ward et al., 2011). To better envisage the translational perspectives of the present study, it is important to include female animals inasmuch that paclitaxel is used in the treatment of tumors that affect women, such as breast, cervical and ovarian cancers (Cavaletti and Marmiroli, 2010).

## Data Availability Statement

The datasets generated for this study are available on request to the corresponding author.

## Ethics Statement

The animal study was reviewed and approved by the Institutional Animal Care and Use Committee of the Faculty of Medicine of the University of Porto.

## Author Contributions

JC-P, IM, and IT participated in study design and planning of experiments, wrote the first versions of the manuscript. JC-P and JR performed the experiments. JC-P, JR, and IM analyzed the data. All authors revised the manuscript.

## Conflict of Interest

The authors declare that the research was conducted in the absence of any commercial or financial relationships that could be construed as a potential conflict of interest.
